# Diagnostic utility of joint fluid metal ion measurement for histopathological findings in metal-on-metal hip replacements

**DOI:** 10.1186/s12891-015-0851-4

**Published:** 2015-12-22

**Authors:** Aleksi Reito, Jyrki Parkkinen, Timo Puolakka, Jorma Pajamäki, Antti Eskelinen

**Affiliations:** Coxa Hospital for Joint Replacement, Biokatu 6b, 33520 Tampere, Finland; Fimlab Laboratories, Tampere, Finland

**Keywords:** Metal-on-metal, Adverse reaction to metal debris, Adverse soft tissue reaction, Joint fluid aspiration, Histology, Synovial response

## Abstract

**Background:**

In vivo assessment of inflammatory responses in the synovia of patients with MoM hip replacements would be useful in the determination of the prognosis of the hip replacement. Aims of the study was to investigate the correlation between cobalt and chrome levels in joint fluid with histopathological findings and the predictive ability of metal ion levels for these findings.

**Methods:**

In 163 revision surgeries (141 ASR THAs and 22 ASR hip resurfacings) joint fluid chrome and cobalt levels were assessed and histological analysis of synovial tissues was performed. Histological analysis included assessment of histiocytes, particle load, surface necrosis, lymphocyte cuffs and ALVAL-score.

**Results:**

Surface necrosis correlated positively with cobalt levels both in both groups. Neither chrome nor cobalt level had even fair discriminative ability to predict the presence or severity of any histological finding in the THA group. In the hip resurfacing group, cobalt level had good discriminative ability to predict the presence of perivascular lymphocytes and ALVAL-score of ≥7 whereas chrome had good discriminative ability to predict surface necrosis, metal particle load and ALVAL-score of ≥7.

**Conclusions:**

Measurement of metal ion levels following joint fluid aspirate offers no relevant information with regard to histopathological findings in patients with large-diameter MoM THAs. Limited information may be gained from assessment of joint fluid metal ion levels in patients with hip resurfacings, but disadvantages of an aspirate must be carefully reviewed.

## Background

Adverse reaction to metal debris (ARMD) continues to be a major issue regarding survival of metal-on-metal (MoM) hip arthroplasties [1–5]. Clinical findings of ARMDARMD often include elevated blood metal ion levels and pain or discomfort in the hip region [6–9]. Sometimes ARMDARMD may go without any clinical findings. Especially in the modular MoM total hip replacements blood metal ion levels may be normal even though clear ARMD is present in the revision surgery [9]. These cases present a diagnostic dilemma. Further issues arise from the fact that natural history of ARMDARMD and pseudotumours, especially cystic pseudotumours, is unknown to a greater extent.

Histopathological findings in failed MoM hip replacements are well described. Patients diagnosed with unexplained pain or ARMD tend to have specific histopathological findings. One entity isALVAL (aseptic lymphocyte-vasculitis associated lesions) which was originally described by Willert and co-workers [10, 11]. ALVAL is not however a pathognomic finding in patients diagnosed with ARMD as shown by Hart et al. [12]. Histiocyte dominated foreign body response is sometimes seen in these patients without any signs of ALVAL [8, 13]. Moreover another study suggests that synovial necrosis with macrophage dominated inflammatory response might be an own entity in some failed MoM hip replacements [14]. In vivo assessment of inflammatory responses in the synovia of patients with MoM hip replacements would therefore be very useful in the determination of the prognosis of the hip replacement. No attempt, however, has been made to examine the correlation of local concentration of metal, ie. joint fluid metal ion level with histopathological findings. Association between concentration and inflammatory response would indicate a threshold of concentration after which ALVAL response is evoked instead of certain temporally associated cumulative dose. Joint fluid metal ion levels may also be more surrogate measurement of wear and metal ion release than levels seen in the periphery (whole blood or serum). Moreover, interpretation of blood metal ion levels is erroneous in patients with bilateral MoM hip arthroplasties in situ. If metal ion levels in the joint fluid are associated with the histopathological findings in the periprosthetic tissues, measurement of these levels would be a potentially effective tool to elucidate the potential status of the implant. Currently, there is no consensus regarding the role of joint fluid aspirations in the diagnostics of ARMD diagnostics.

We aimed to study the etiological factors of synovial inflammatory response and diagnostic utility of measurement of metal ion levels after joint fluid aspiration. Primary aim of the study was to investigate whether there is correlation between cobalt and chrome levels in joint fluid with histopathological findings seen in the synovia of patients with failed MoM hip replacement. Secondary aim was to investigate the discriminative ability of joint fluid metal ion levels predicting histopathological findings in these patients.

## Methods

This was a level II study with development of diagnostic criteria on basis of consecutively operated patients with applied gold standard (histological analysis of synovia retrieved in revision surgery). One thousand thirty-six Anatomic Surface Replacement (ASR) THA (US Food and Drug Administration, FDA, approved) or total hip resurfacing arthroplasty (FDA not approved) (DePuy, Warsaw, IN) were performed consecutively in 887 patients at our institution between March 2004 and December 2009. After the ASR recall in August 2010, we established an intensified screening program for all patients with ASR MoM hips [15]. All patients have undergone intense work-up including clinical examination, whole blood cobalt and chrome metal ion measurement and cross-sectional imaging [2]. Revision surgery was considered and suggested to patient if (1) there was a clear pseudotumor (Imperial class 2A, 2B or 3) observed on cross-sectional imaging regardless of symptoms or whole blood metal ion levels; or (2) the patient had elevated whole blood metal ion levels and hip symptoms despite a normal finding on cross-sectional imaging; or (3) the patient had a continuously symptomatic hip or progressive symptoms regardless of imaging findings or metal ion levels [12]. Symptoms included hip pain, discomfort, sense of instability, and/or impaired function of the hip and sounds from the hip (clacking, squeaking). Whole blood metal ion levels were considered elevated if either chromium or cobalt exceeded 5 ppb [16].

For the purposes of this study we identified all patients who have undergone revision surgery of the MoM hip because of ARMD at our institution. Diagnosis of ARMD was based on perioperative findings. Failure was classified as being secondary to ARMD if the following, previously published criteria were met: (1) there was either metallosis or macroscopic synovitis in the hip joint; and/or (2) a pseudotumor was found during revision; and/or (3) a moderate to high amount of perivascular lymphocytes along with tissue necrosis and/or fibrin deposition was seen in the histopathologic sample; and (4) perioperatively there was no evidence of component loosening or periprosthetic fracture [2]. Furthermore, infection was ruled out by multiple (at least five) bacterial cultures obtained during revision surgery.

### Study group

**Fig. 1:**
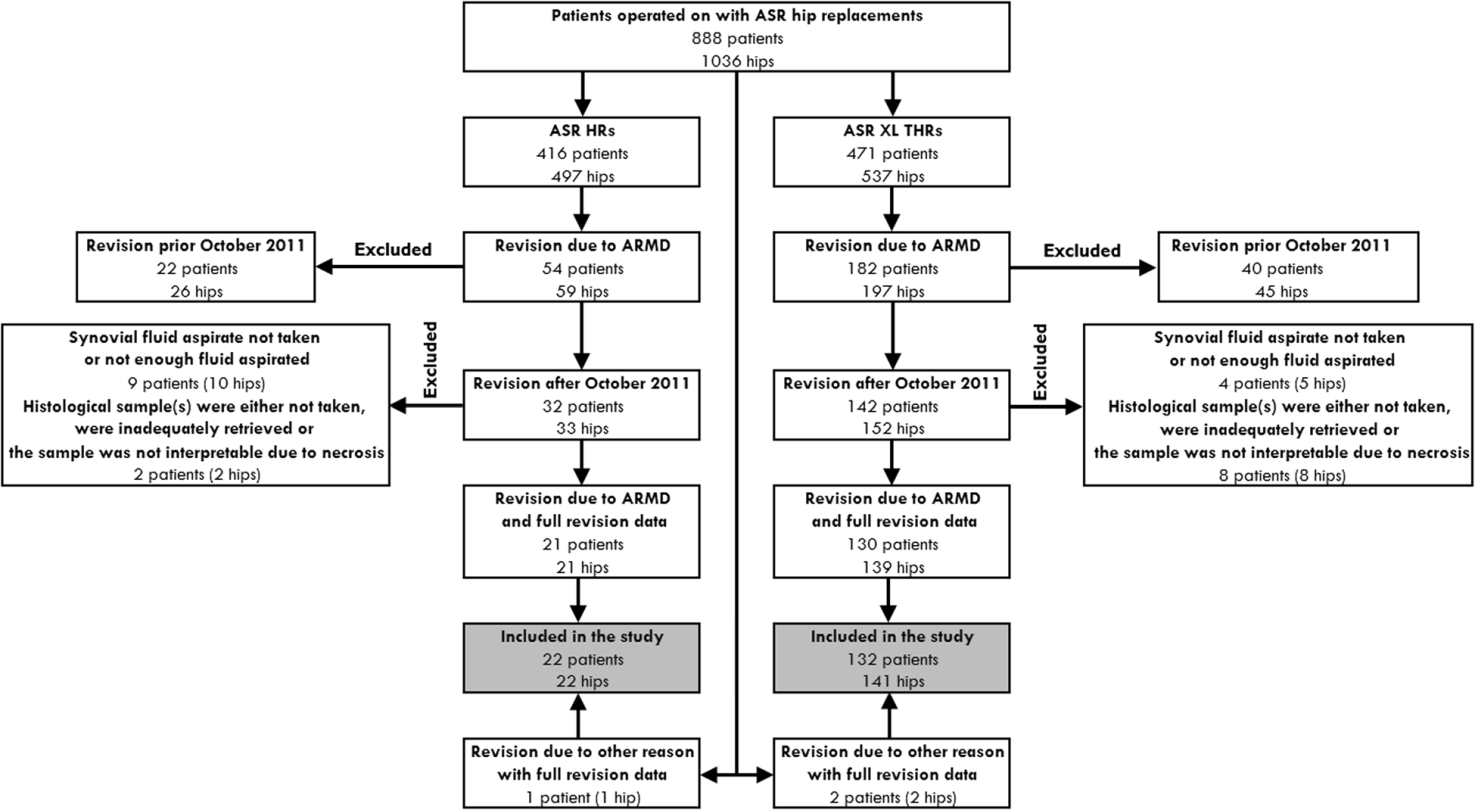
Flow chart of the study group selection

Flow hart of the patient selection is shown in Fig. 1. In total 256 hips have been revised due to ARMD. The prevalence of ARMD was significantly higher in the THR group compared to HR group (36.7 % vs. 11.8 %, *p* < 0.001). Since October 2011, ARMDthere have been 185 revision operations in 174 patients due to ARMD. Both all primary operations and revision operations of these patients were performed at our institution. In none of these 185 hips were there any signs of component loosening perioperatively, neither were there positive findings in bacterial cultures, and macroscopic findings of ARMD were present in each of these hips. In 10 (4.7 %) hips, histological sample(s) were either not taken, were inadequately retrieved or the sample was not interpretable due to necrosis. Further, in 15 (11 %) hips the synovial fluid aspirate was either not taken or not enough fluid was aspirated. This left 160 of the 185 hips revised due to ARMD for analysis. In addition, synovial aspirate was performed and synovia sample analyzed in three hips of which one was revised for aseptic loosening of the stem and two for unexplained pain. The case with aseptic loosening had macroscopic findings indicating also presence of ARMD. Two cases with unexplained pain had only very mild metallotic staining and diagnosis of ARMD could not be readily done. Thus, 163 hips were included in the study group, which consisted of two subgroups: (1) The hip resurfacing group, which included 22 hips (22 patients), and (2) the THA group, which included 141 hips (132 patients). A written informed consent was obtained from all patients participating in this study. We obtained permission to perform this study from the ethics committee (Regional Ethics Committee in The Pirkanmaa Hospital District’s Science Centre) of the hospital district in which the study was conducted.

### Revisions and revision protocol

Six orthopaedic surgeons (including co-authors JP and TP) performed revisions using posterior approach. Techniques and implants used in these revision surgeries have been described in detail in our recent study [17]. In each revision operation, 1–5 samples of synovia and/or pseudotumour tissue were taken for analysis. Primary site of the sample was pseudotumour capsule, and if such tissue was not present, sample was taken from the pseudocapsule.

### Joint fluid aspirate

Since October 2011, our perioperative MoM hip revision protocol has involved joint fluid aspiration, which is always taken before opening the deep fascia using a standard 18–20 gauge needle connected to a Vacutainer system (Becton, Dickinson and Company, Franklin Lakes, New Jersey) and trace element tubes containing sodium ethylene diamine tetraacetic acid (EDTA).

### Measurement of metal ion levels

In the Finnish Institute for Occupational Health, standard operating procedures were established for Co and Cr measurement using dynamic reaction cell inductively coupled plasma (quadripole) mass spectrometry (Agilent 7500 cx, Agilent Technologies, Santa Clara, CA, USA). The laboratory technicians were blinded to all clinical outcomes. The samples were preserved in +6–+8 °C prior to analysis. Samples were tested using detection limits (DL) similar to those with whole blood sample. DL for Cr was of 0.03 ppb and for Co of 0.3 ppb.

### Histology

Each tissue sample was formalin fixed. The samples were embedded in paraffin and several 10 μm microtome sections were made. Standard hematoxylin and eosin staining was used. The sections were examined histologically under normal light Nikon Eclipse 50i (Nikon Corporation, Shinagawa, Tokyo, Japan). For investigational purposes all available samples were retrospectively analyzed according to principles described by Natu et al. [11]. Necrosis was classified from Grade I to IV according to Natu et al. Natu grading of necrosis is based to loss in synovial integrity described by Davies et al. [18]. Davies Type 1 synovial surface is intact epithelium. Type 2 synovial surface has loss of cellular lining but without fibrin exudation. Type 3 surface has both loss of synovial cell lining and fibrin exudate is present. Type 4 synovial surface has gross disruption, fissuring and fibrin exudates. In Natu Grade I necrosis synovial consisted of only Davies Type 1 or 2. Grade II consisted of Davies Type 3 or maximally 25 % of Type 4. In Grade III necrosis surface consisted between 25 % and 75 % of Davies Type IV. In Grade IV surface showed more than 75 % of Davies Type IV synovial loss. Lymphocytic cuff thickness was calculated using graticule. An average of five measurements was taken and graded as 0–3 (absent, 0.25 mm, 0.25 – 0.75 mm, >0.75 mm). Thickness of histiocyte sheets was calculated and graded 0–3 (absent, <1 mm, 1 – 2 mm, >2 mm). Particle load within histiocytes was graded as used in the assessment of iron decomposition in liver cells [11]. In addition to these analyses ALVAL-score was also assessed in each sample [13].

### Statistics

Summary of statistical methods and their implemented interpretation in relation research questions is shown in Table 1. This was a Level III study. Due to retrospective nature of our study sample size assessment was not performed and instead the study was conducted including all available hips for analyses. However, sample size calculation was done afterwards in a post-hoc manner in order to investigate the power of our analysis as recommended by the STROBE guidelines [19]. Receiver operator characteristics (ROC) can be used to investigate the performance of diagnostic continuous variable against binary classifier. We considered area under curve (AUC) ≥ .75 in the ROC curve as clinically useful test [20].

**Table 1:** Scheme of methods of present study

Research question	Statistical method	Outcome
1) Is there association between joint fluid metal ion levels with histological observations?	Spearman rank correlation coefficient	Higher value of significant coefficient indicates higher association with metal ion levels and histological findings
2) Do cobalt and chrome levels in joint offer good or excellent discriminative ability predicting histological findings?	Value and significance of AUC in ROC curve using Obuchowski method	AUC of >0.75 indicates good or excellent AUC. Sample size criterion must be met to produce *significant* AUC
3) What is the sensitivity and specificity of optimal cut-off value for chrome and cobalt levels predicting histopathological findings?	Youden J -statistic	Produces optimal threshold (or cut-off) value defined as *maximal distance* from ROC curve to diagonal line

Distribution of joint fluid cobalt and chrome values were highly skewed to left when inspecting histograms thus implying violation of assumption of normality. This was confirmed with Kolmogorov-Smirnov test with Lilliefors correction (Cr: *p* = .8, Co: *p* = .8). Mann–Whitney U -test was when comparing metal ion levels in joint fluid when two groups were available. Kruskal-Wallis test was used with more than two group comparisons. Spearman rank correlation was used to study the association of ordinal observation and joint fluid metal ion levels. Since the distribution of ALVAL-score is known we treated it both categorical and continuous variable and hence calculated both Kruskal-Wallis statistics and correlation coefficient when examining the association between ALVAL-score and metal ion levels. ROC analysis was performed to study the discriminative ability of cobalt and chrome levels in joint fluid for each histological observation. Due to lack of clinically relevant cut-off values for observation with more than two classes (ie. thickness of lymphocyte cuffs) AUC was calculated using each possible subclass as threshold only if a sample size criterion was fulfilled. Finally, sensitivity and specificity was calculated and interpreted only if AUC seen in the ROC curve was >0.75 and AUC was also statistically significant as described earlier. In the assessment of sensitivity and specificity Youden J -statistic was used. This method offers the optimal cut-off value indicating the threshold that maximizes the distance to the identity (diagonal) line.

**Fig. 2:**
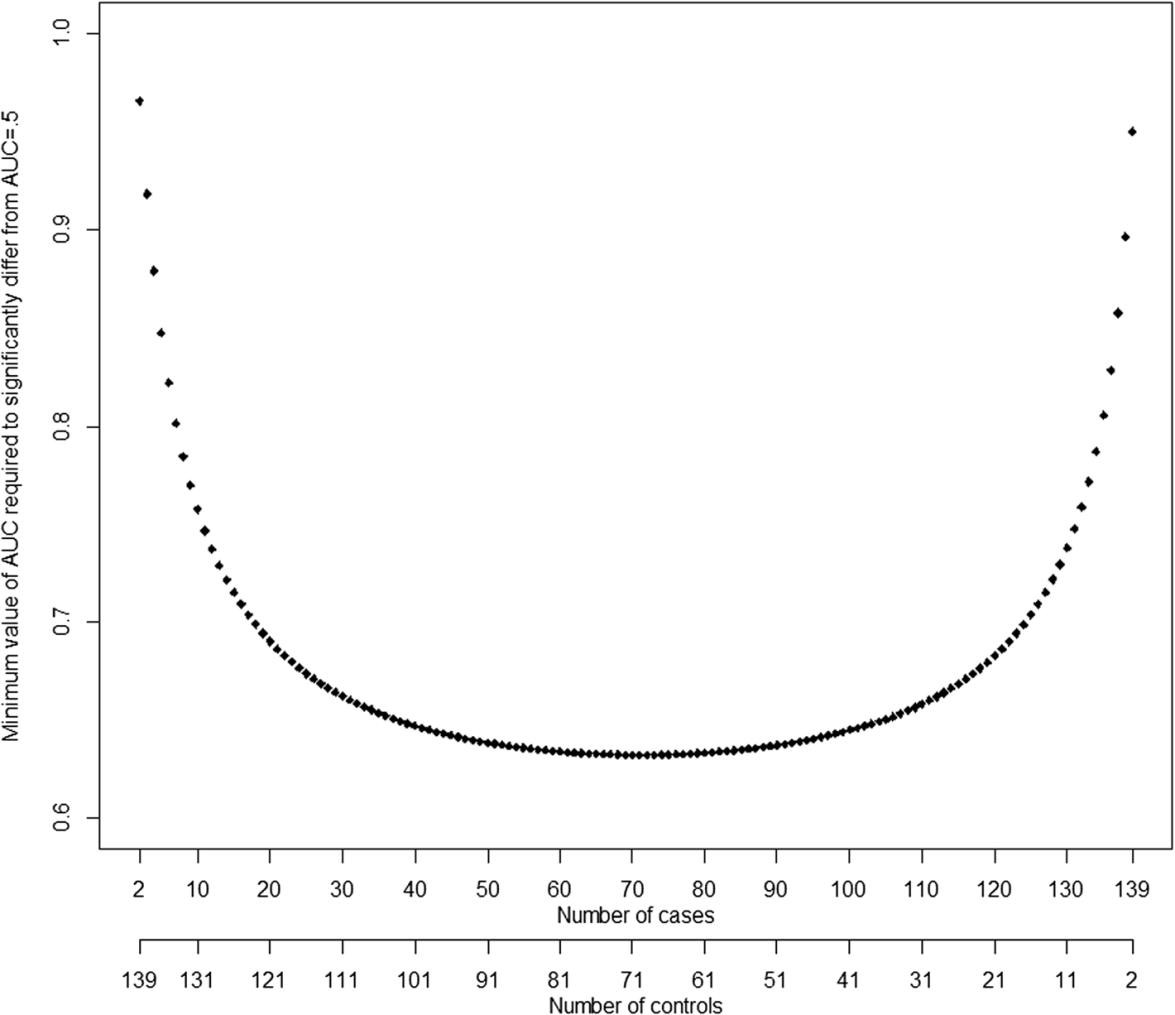
Interpretation of required sample size in order to have significant AUC at level .05 with power .80 with 141 hips in the THA group

**Fig. 3:**
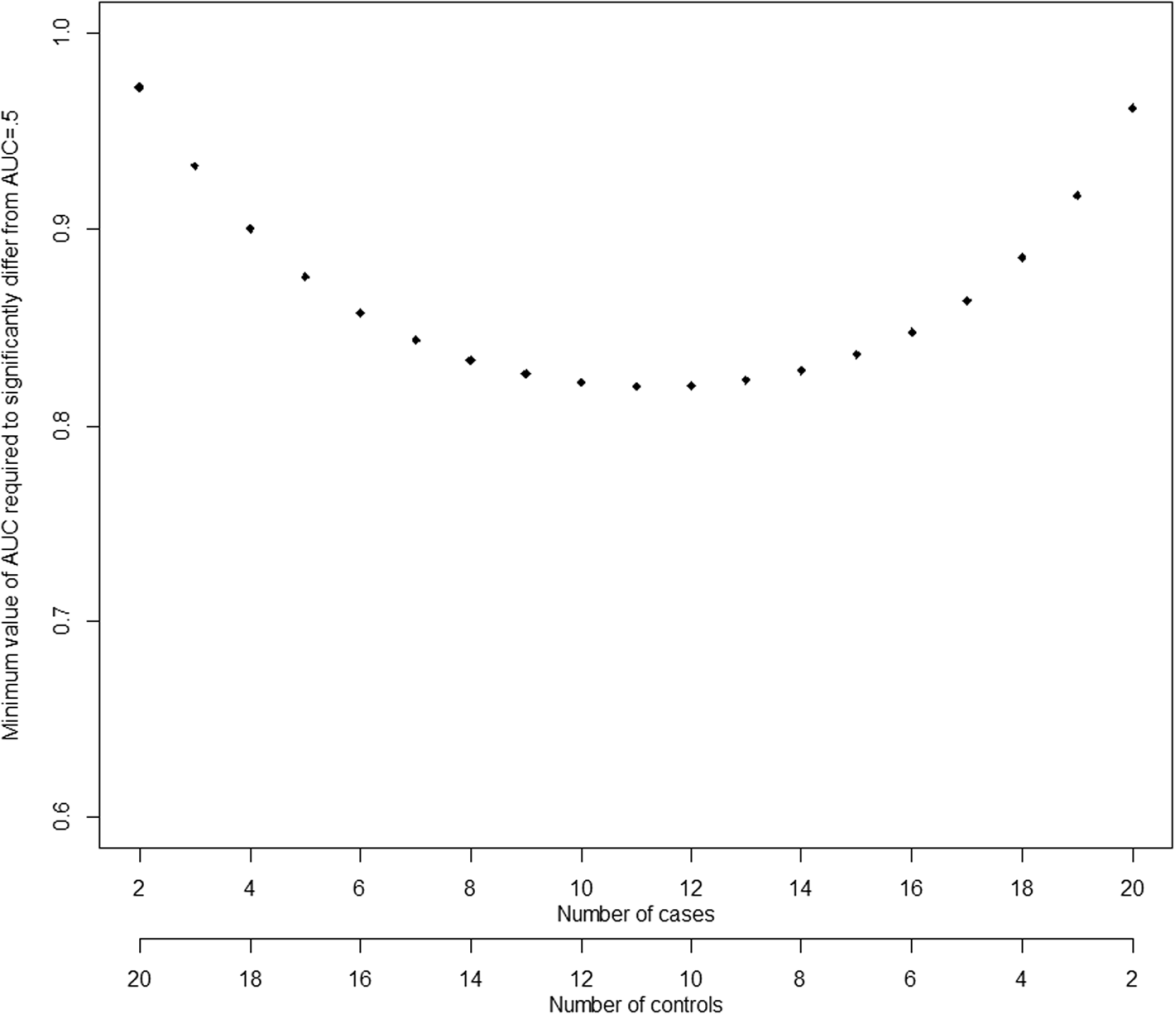
Interpretation of required sample size in order to have significant AUC at level .05 with power .80 with 22 hips in the hip resurfacing group

We performed post-hoc power calculation as described by Obuchowski et al. [21]. When performing sample size estimation for ROC analysis one hypotheses that estimated AUC differs significantly (at level .05) from AUC of 0.5 (ie. “better than coin flip”). Calculation requires that significance level (Type I error) and study power (1 – Type II error) are known. The former was set to .05 (5 %) and the latter to .80 (80 %), which are the most commonly used values. In addition, the ratio of controls versus cases is required for the analyses. Since our analysis involves several different study settings, one specific set of required number of cases and control was not calculated. Instead required number controls and cases were calculated based on available kappa for each ROC analysis separately. Graph implying the required number cases and control was drawn separately for hip resurfacings and THAs (Figs. 2 and 3). Interpretation of sample size is assessed as follows: total number THAs was 141. Hence sum of controls (hips without a desired observation) and cases (hips with desired observation) equals always 141 as shown in dual X-axis in Fig. 2. If we had 20 hips with desired end-point (cases), there would be 121 hips as controls. In order to have significantly better discriminative ability with power of .80 for AUC compared to AUC = 0.5 (“better than coin flip)”, an AUC of 0.70 is needed in ROC analysis. As a general rule as seen in the graphs, the higher the number of cases and controls or the ratio of controls:cases, the smaller AUC is required to be significantly better than AUC = 0.5. Significance was set to .05. Statistical analysis was performed with R 3.0.2 using pROC package.

## Results

**Fig. 4:**
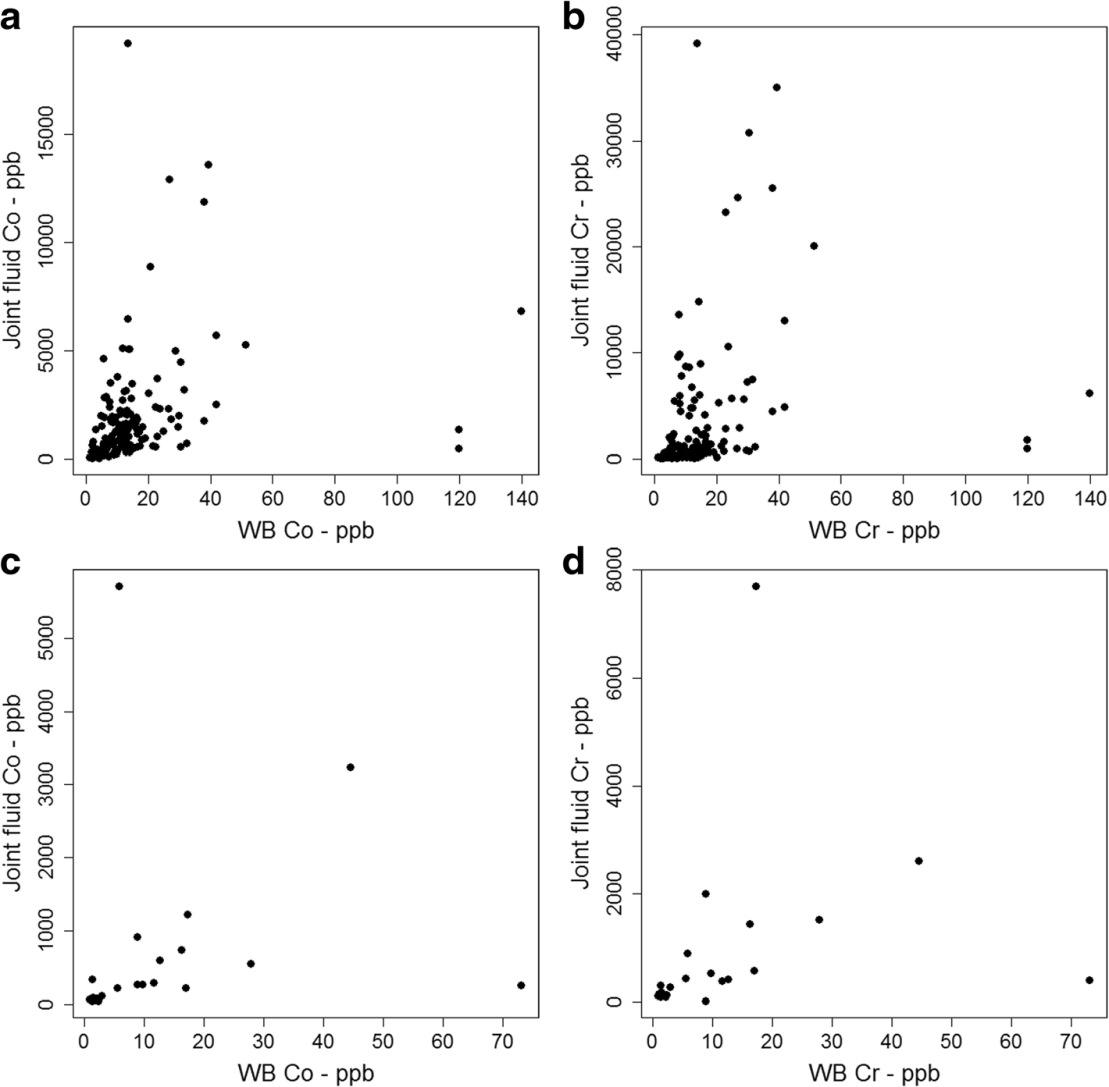
Scatter plots of joint fluid versus WB metal ion levels. **a** Scatter plot of joint fluid cobalt versus WB cobalt in THAs. **b** Scatter plot of joint fluid chrome versus WB chrome in THRs. **c** Scatter plot of joint fluid cobalt versus WB cobalt in hip resurfacings. **d** Scatter plot of joint fluid chrome versus WB chrome in hip resurfacings

Comparison of demographical, clinical and histological variables between groups is shown in Table 2. There was significant correlation between joint fluid and whole blood metal ion levels in both implant groups (Fig. 4). Strength of correlation, however, was poorer in the THA (Cr: ρ = .525, *p* < .001; Co: ρ = .487, *p* < .001) than in the hip resurfacing group (Cr: ρ = .727, *p* < .001; Co: ρ = .602, *p* < .001).

**Table 2:** Demographics, metal ion levels and frequencies of histological findings distributed by implant type

			THA	Hip resurfacing	*p*-value
Demographic variables	Age	Mean (SD)	60.5 years (8.4)	50.4 years (8.6)	<.001
	Gender	Males Females	72 69	12 10	.9
Metal ion levels	Whole blood	Median Co level (range)	11.9 ppb (1.1 – 139.9)	7.35 ppb (1 – 73)	.03
		Median Cr level (range)	3.4 ppb (0.7 – 61.6)	3.85 ppb (0.8 – 31)	.4
	Joint fluid	Median Co level (range)	1291 ppb (36 – 19150)	271 ppb (40–5702)	<.001
		Median Cr level (range)	903 ppb (24 – 39160)	390 ppb (4 ppb – 7698)	.004
Histological observations	Lymphocyte cuff thickness	Absent	50	10	.3
		<0.25 mm	81	12	
		0.25 – 0.75 mm	10	-	
	Histiocyte sheet thickness	Absent	4	-	.4
		<1 mm	108	18	
		1–2 mm	24	2	
		>2 mm	5	2	
	Surface necrosis	Grade I	7	9	<.001
		Grade II	34	4	
		Grade III	38	7	
		Grade IV	62	2	
	Particle load	Grade 0	28	4	.6
		Grade 1	25	5	
		Grade 2	37	3	
		Grade 3	36	6	
		Grade 4	15	4

**Fig. 5:**
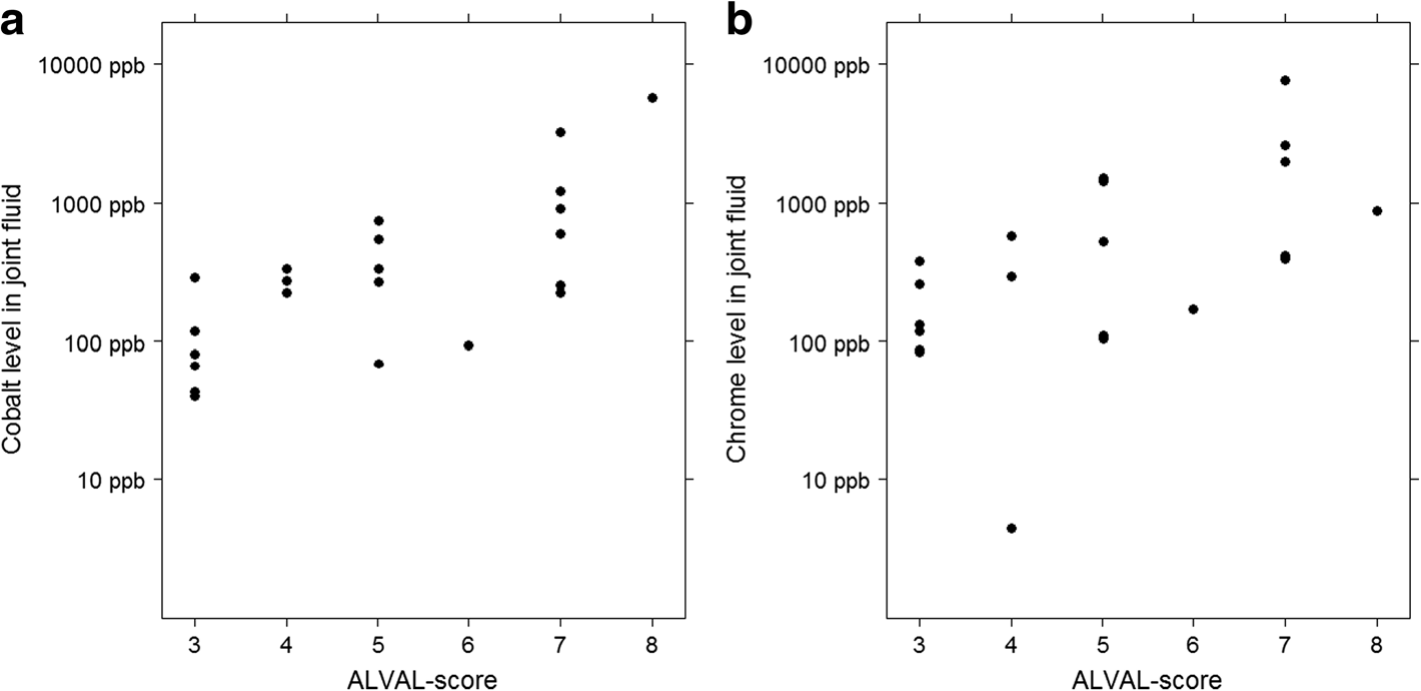
Scatter plot of ALVAL-score versus joint fluid metal ion levels. **a** Scatter plot of cobalt level in joint fluid versus ALVAL-score in the THA group. **b** Scatter plot of chrome level in joint fluid versus ALVAL-score in the hip resurfacing group

In the THA group cobalt levels correlated positively with the surface necrosis whereas chrome levels did not (Table 3). Median chrome and cobalt levels were different across different grades of surface necrosis in the hip resurfacing group. Associative statistics revealed that both chrome and cobalt correlated positively with the severity of surface necrosis. No other differences of correlations were seen in the THA group (Table 3). In the hip resurfacing group, there were no hips with lymphocyte cuff of >0.25 mm, and hence two group comparisons was used (Tables 2 and 3). Hips without lymphocyte cuff had lower median chrome and cobalt levels compared to hips with cuff thickness of 0–0.25 mm (Cr: 474 ppb vs. 119 ppb, Co: 466 ppb vs. 88 ppb). There was also a positive correlation with lymphocyte cuff thickness and chrome and cobalt joint fluid levels. Neither did ALVAL-score correlate with joint fluid chrome or cobalt level in the THA group (Co: *p* = .2, Cr: *p* = .6). In the hip resurfacings group, both joint fluid chrome and cobalt correlated positively with ALVAL-score (Co: *p* < .001, Effect size: ρ = .675; Cr: *p* = .001, Effect size: ρ = .641) (Fig. 5).

**Table 3:** Differences and correlations of synovial metal ion levels in respective to ordinal histological observations

		THA		Hip resurfacing	
		Chrome	Cobalt	Chrome	Cobalt
Surface necrosis	Correlation	*p* = .3	*p* = .009 Effect size: *ρ* = .21	*p* = .014 Effect size: *ρ* = .511	*p* = .002 Effect size: *ρ* = .632
Thickness of histiocyte sheets	Correlation	*p* = .2	*p* = .5	*p* = .2	*p* = .2
Thickness of lymphocyte cuffs	Correlation	*p* = .3	*p* = .4	*p* = .008 Effect size: *ρ* = .546	*p* = .001 Effect size: *ρ* = .640
Particle load	Correlation	*p* = .9	*p* = .2	*p* = .4	*p* = .7

Neither chrome nor cobalt level had even fair discriminative ability to predict the presence or severity of any histological finding in the THA group. Only one significant AUC (> AUC = .5) was seen in the THA group (Tables 4 and 5). However, this AUC predicting ALVAL score of 5 or higher was so low that it indicated poor discriminative ability. In the hip resurfacing group, several useful AUCs with good or excellent discriminative ability were seen (Tables 4 and 5). In this group, cobalt level alone had good discriminative ability to predict the presence of lymphocyte cuffs (>0 mm), Grade II or more severe surface necrosis and grade 1 or higher particle load. Both chrome and cobalt level had good discriminative ability to predict ALVAL-score of ≥7 (Table 5). There was not enough cases to use ALVAL score of ≥8 or ≥9 as an end-point.

**Table 4:** AUCs for ordinal observations with all possible predictive values

		THA		Hip resurfacing	
Observation	Predictive value	Joint fluid Chrome AUC	Joint fluid Cobalt AUC	Joint fluid Chrome AUC	Joint fluid Cobalt AUC
Lymphocyte cuff thickness	>0 mm	.549	.476	.817	.871^a^
	>0.25 mm	.538	.623	n/a	n/a
Histiocyte cuff thickness	>0 mm	.586	.467	n/a	n/a
	≥1 mm	.523	.606	.736	.687
Surface necrosis	Grade II-IV	.551	.688	.709	.829^a^
	Grade III-IV	.529	.621	.820	.807
	Grade IV	.638	.582	.85	.95
Particle load	Grade 1–4	.536	.618	.638	.895^a^
	Grade 2–4	.492	.571	.552	.625
	Grade 3–4	.480	.489	.495	.551
	Grade 4	.496	.504	.588	.677

*n/a* assessment of AUC not possible due to inadequate number of controls and cases, see Table 1. ^a^ = significantly different from AUC = .5, see Figs. 2 and 3

**Table 5:** AUCs for ALVAL-scores with all possible predictive values

		THA		Hip resurfacing	
ALVAL-score as predictive value		Joint fluid chrome AUC	Joint fluid cobalt AUC	Joint fluid chrome AUC	Joint fluid cobalt AUC
≥4	Low	.633	.649	.823	.867
≥5	Moderate	.709^a^	.620	.829	.821
≥6		.511	.593	.813	.781
≥7		.546	.479	.867^a^	.852^a^
≥8		.575	.510	n/a	n/a
≥9	High	.597	.478	n/a	n/a

*n/a* assessment of AUC not possible due to inadequate number of controls and cases, see Table 1. ^a^ = significantly different from AUC = .5, see Figs. 1 and 2

**Fig. 6:**
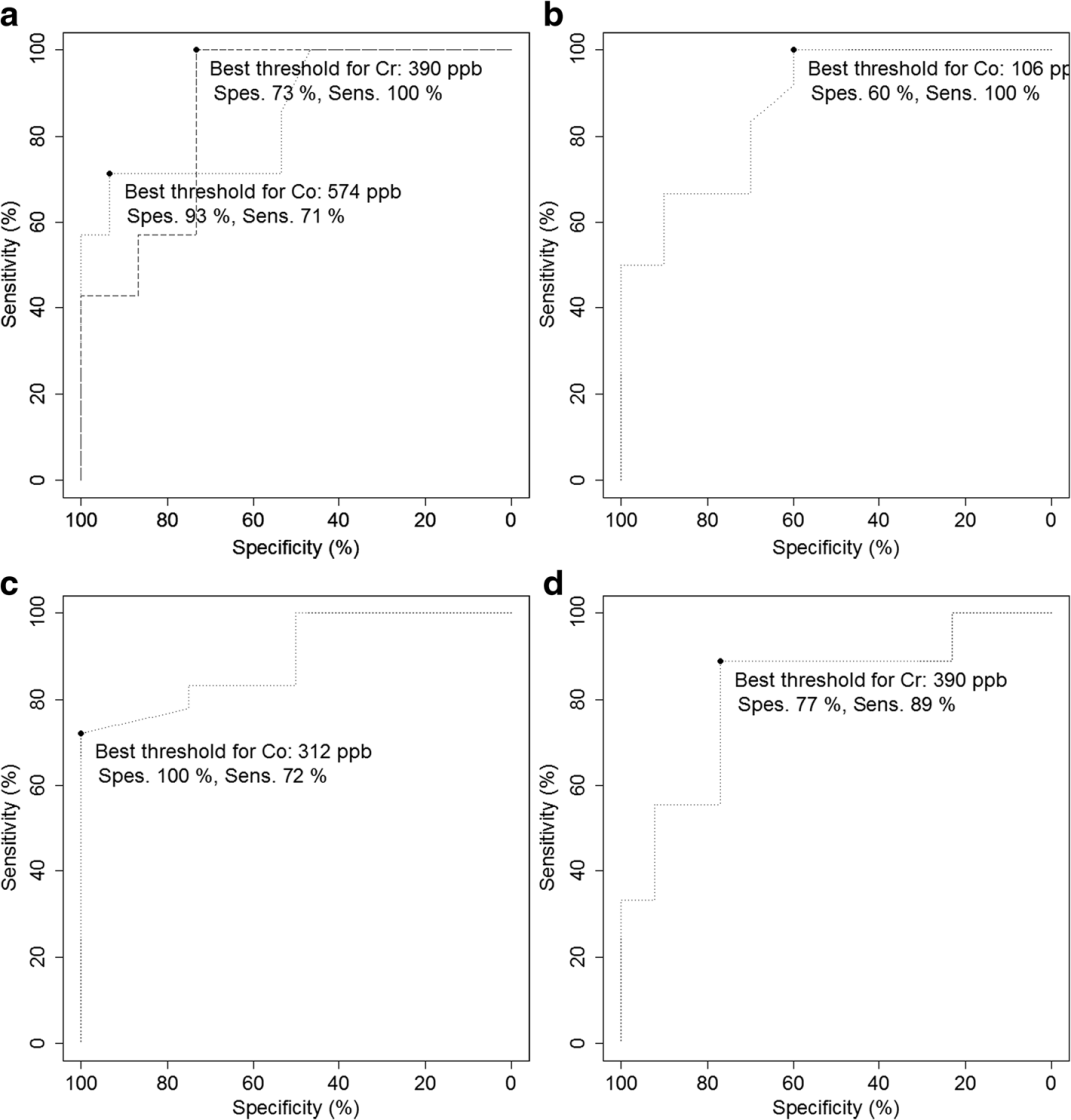
ROC curve and best threshold with corresponding value for sensitivity and specificity for each outcome with significant AUC of > .75 in the hip resurfacing group. **a** Joint fluid Co and Cr level predicting ALVAL-score of 7 or higher. **b** Joint fluid Co level predicting presence of lymphocyte cuffs. **c** Joint fluid Co predicting particle load graded 1–4. **d** Joint fluid Cr predicting grade III-IV surface necrosis

Chrome and cobalt levels in joint fluid had good discriminative ability to predict five different histopathological findings, but only in the hip resurfacing group (Tables 4 and 5). Values for best sensitivity and specificity were assessed in these five cases (Fig. 6).

## Discussion

Identification of ARMD is a challenging process. Although a wide range of novel methods are available, especially whole blood metal ion measurement and cross-sectional imaging, interpretation of results obtained by these methods presents a diagnostic dilemma in some cases. Extremities of findings do not introduce a diagnostic problem: a large, thick walled pseudotumour with atypical content or extremely elevated blood metal ion levels are clear signs of poorly functioning MoM hip. However there is a wide range of findings, e.g. slightly elevated blood metal ions and/or small cystic pseudotumour seen in cross-sectional imaging, that present a diagnostic problem. Correlation of histopathological findings with bearing wear and tissue metal content have been reported earlier [22, 23]. This kind of quantitative data is however obtained only after revision operation. Whole blood metal ion levels has been proposed being a surrogate measurement for bearing and hardware wear but results with regard to this topic is very controversial [24]. Both total hardware wear and tissue metal content are matters of a cumulative dose. There are no current recommendations on how to interpret the results of joint fluid metal ion measurements. There is also relative paucity in the current literature with regard to usefulness of joint fluid metal ion measurements.

We acknowledge some limitations in our study. First, there may be selection bias in our material. All except three of our patients were diagnosed with ARMD indicating that there was lack of hips with unresponsive synovium (e.g. patients revised for periprosthetic fracture or implant loosening). Including such hips would have increased the strength of our study, since all hips in our study group had some histopathological findings. On the other hand, hips with presumably unresponsive synovia are not usually scheduled to undergo synovial aspiration and revision surgery, and therefore our study group is a good sample of clinically relevant hips. Second, our study group does not directly reflect the population that normally undergoes synovial aspiration as part of diagnostic work-up for unexplained pain or for suspected ARMD. Instead, all of our patients had already been listed for revision. However, collecting our samples perioperatively enabled us to have optimal circumstances for a good and representative fluid aspirate. Third, perioperative joint aspiration was not used in the early revisions. This presents a selection bias in the study analyses. Our study cohort may lack the most severe cases of ARMD assuming they had undergone revision surgery prior to utilizing routine joint aspiration procedure. However, the cause of this bias cannot be reliably assessed since the association between wear and histopathology is not clearly established. Finally, samples obtained from soft tissues perioperatively might not represent the overall response of the synovia. It is not known to what extent one sample of synovia present the actual type of histopathology in each case. Several samples would minimize this variation but this approach has practical limitations since tissue preservation is important during revision surgery. Furthermore, there might be considerable variation of cell counts among different sections from same sample. However we think that this sampling bias is reduced significantly due to large group of cases.

Several authors have reported joint fluid metal ion levels in failed MoM hip replacements [8, 25, 26]. Kwon et al. compared joint fluid metal ion levels in five patients with pseudotumour and 13 patients without one [25]. There was practically no overlapping in metal ion levels. Co levels ranged between 206–1802 in patients with pseudotumour compared to range of 1.0–158 ppb in patients without a pseudotumour. Respective Cr values were 221–1322 ppb and 3–230 ppb. As so Co level of >206 ppb and Cr level >221 ppb had very high sensitivity and specificity for predicting the presence of a pseudotumour. Majority of pseudotumours in that study were cystic ones, which are not necessarily always related to ARMD. Cystic fluid collections are also seen in non-MoM hip replacements [27]. Interestingly, however, threshold levels seen in the study by Kwon et al. are below our threshold values for adverse synovial responses. As a novel finding, we found that when joint fluid ion levels exceed the threshold levels established by Kwon et al. the possibility of synovial necrosis and ALVAL-score >7 becomes more likely, possible indicating presence of ALVAL response in the synovia and mixed/solid pseudotumour. If progression of ARMD were this straightforward, assessment of joint fluid metal ion levels would be of great relevance.

Both in the hip resurfacing and in the THA groups, severe surface necrosis was associated to high cobalt levels in the joint fluid. This might imply a direct cytotoxic effect of cobalt ions as proposed by Mahendra et al. [14]. Interestingly, we observed higher metal ion levels in hips with lymphocyte cuffs in the hip resurfacing group. This result concurs with Lohmann et al. who stated that perivascular lymphocyte infiltration was more prevalent in hips with high tissue metal content [23]. Since necrosis, lymphocyte cuffs and loss in cell structure is characteristic for severe ALVAL, this result suggest that ALVAL might be a dose-dependent finding, at least in patients with hip resurfacings. It also brings up an important question: does hypersensitivity or metal allergy truly exist. This issue certainly warrants further research. It is, however, unclear why perivascular lymphocytes had no association with metal ion levels in the THA group. Since only necrosis was associated to cobalt ion levels in the joint fluid of patients with failed THA, a direct cytotoxic effect might be more prevalent response in this group. Other authors have previously suggested that debris originating from the taper might be more toxic than that originating from the bearing surface [28]. Our results indirectly support this hypothesis.

In the hip resurfacing group, sensitivity and specificity of cobalt and chrome levels in joint fluid for predicting five different histological finding was relatively high. These results suggest that the assessment of metal ion levels from the joint fluid may be a useful clinical tool in patients with symptomatic hip resurfacings, or in such patients with slightly elevated blood metal ions. Assessment of joint fluid metal ion levels and synovial inflammatory response would be especially useful, if a patient with hip resurfacing has elevated blood metal ions or hip symptoms, but no pseudotumor is seen in cross-sectional imaging. In such cases, joint fluid metal ion levels may give additional information on the synovial response to metal debris. Especially low synovial metal ion levels can confirm that there is no synovial response in that hip. Another situation, where joint fluid metal ion assessment can be useful, is when an asymptomatic patient with bilateral MoM hips has markedly elevated blood metal ions. In such situation, especially in the absence of any findings in cross-sectional imaging, joint fluid aspirate may help in identifying, whether only one or both of the hips are excessively producing metal debris. It is important to note, however, that in our material the values for the best threshold varied a lot, possibly highlighting the independency of each histopathological observation. Thus the interpretation of the joint fluid levels must be done with caution especially within the range from 109 ppb to 574 ppb.

## Conclusion

We found that in patients with failed MoM hip replacement the histopathological findings had relatively poor correlation with the chrome and cobalt levels in the joint fluid. This was especially true in the THA group. We consider this a worrisome finding since this indicates that non-bearing surface wear debris may have a direct cytotoxic effect on synovia. In patients with hip resurfacings, on the contrary, joint fluid metal ion levels had at least a fair discriminative ability to predict surface necrosis and high ALVAL-score. This finding suggests a non-linear dose-dependency for metal ions produced by the bearing surface, ie. there may be a certain threshold for these metal ions to launch a lymphocyte-dominated inflammatory response. Further studies with larger sample size are required to confirm our preliminary results. Based on these results, however, it seems to be clear that routine measurement of joint fluid metal ion levels in patients with MoM hips are neither useful nor advisable in order to predict the synovial inflammatory responses.

## Abbreviations

ARMD: Adverse reaction to metal debris
ALVAL: Aseptic lymphocyte-vasculitis associated lesions
AUC: Area under curve
THA: Total hip arthroplasty
